# Repurposing of drugs targeting the cytokine storm induced by SARS‐CoV‐2

**DOI:** 10.1111/bph.15987

**Published:** 2022-11-30

**Authors:** Wern Hann Ng, Patrick Chun Hean Tang, Suresh Mahalingam, Xiang Liu

**Affiliations:** ^1^ Emerging Viruses, Inflammation and Therapeutics Group, Menzies Health Institute Queensland Griffith University Gold Coast QLD Australia; ^2^ Global Virus Network (GVN) Centre of Excellence in Arboviruses Griffith University Gold Coast QLD Australia; ^3^ School of Pharmacy and Medical Sciences Griffith University Gold Coast QLD Australia

**Keywords:** COVID‐19, cytokine storm, immunosuppressive drugs, SARS‐CoV‐2

## Abstract

A cytokine storm is one of the leading causes of acute respiratory distress syndrome (ARDS) and sepsis‐associated multiple organ failure in many respiratory viral infections, including severe acute respiratory syndrome coronavirus 2 (SARS‐CoV‐2). The coronavirus disease 2019 (COVID‐19) pandemic has caused millions of deaths worldwide, resulting in an urgent need for effective therapeutic interventions. Repurposing immunosuppressive drugs that target cytokines with immunomodulatory properties is a promising approach to counteract SARS‐CoV‐2‐induced ARDS at the infective and post‐infective stages. In this minireview, we examine drugs targeting IL‐1β, IL‐4/IL‐13, IL‐6 and TNF‐α tested in COVID‐19 patients.

## 1 INTRODUCTION

**FIGURE 1:**
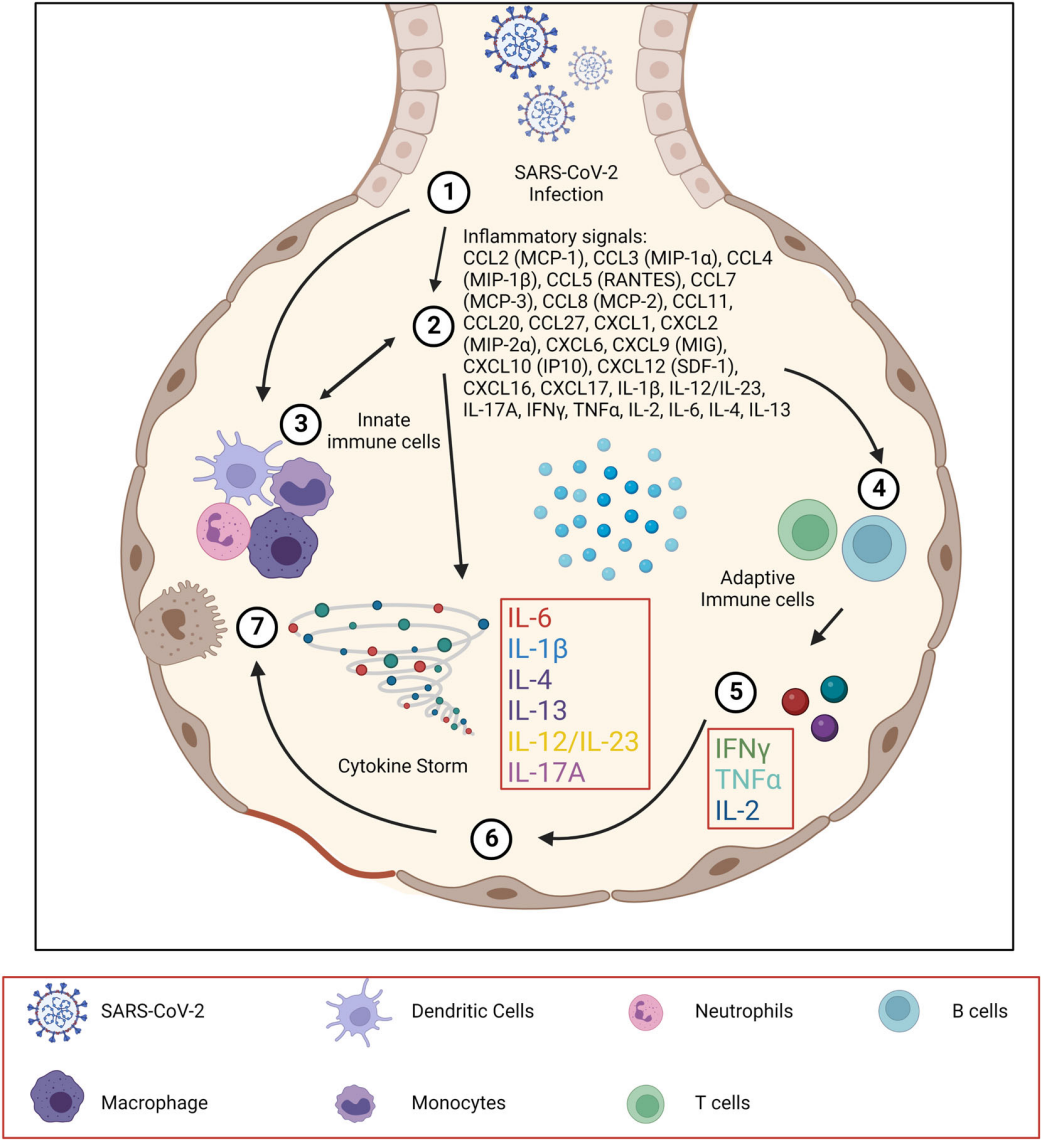
Cytokine storm in COVID‐19. SARS‐CoV‐2 infection of lung airway epithelial cells results in robust proinflammatory cytokine production, leading to severe inflammation (recruitment of immune cells) and subsequent respiratory failure. Recruited cells (monocytes, neutrophils, T cells) produce proinflammatory cytokines, further amplifying the inflammatory response.

The coronavirus disease 2019 (COVID‐19) pandemic caused by severe acute respiratory syndrome coronavirus 2 (SARS‐CoV‐2) has resulted in more than 619 million confirmed cases and 6.5 million deaths worldwide, with the total death (“excess mortality”) associated directly or indirectly with the pandemic, at approximately 14.9 million. Immunopathology, including lymphopenia, neutrophilia, dysregulation of monocytes and macrophages and hypercytokinemia, also known as a cytokine storm, may be largely responsible for the pathogenesis of COVID‐19 (Hu et al., 2021). In its most severe form, the disease can lead to acute respiratory distress syndrome (ARDS), multiple organ failure and death (Ye et al., 2020). Several proinflammatory cytokines and chemokines have been identified in patients with COVID‐19 (Chen et al., 2020; Huang et al., 2020; Vaninov, 2020) (Figure 1). Elevated levels of these cytokines in the lung alveoli have been associated with mortality in patients diagnosed with ARDS (Huang et al., 2020). In severe cases, lung scarring may occur, resulting in lung tissue stiffness, long‐term disability and even death owing to lung fibrosis. Additionally, local cytokine storms can spread to other body parts, leading to sepsis. Therefore, early clinical intervention against cytokine storms in COVID‐19 patients is essential to prevent morbidity and mortality (Cappanera et al., 2021).

The global spread of SARS‐CoV‐2 highlights the urgent need for effective therapeutic strategies. Anti‐cytokine drugs have been used extensively to treat inflammatory and autoimmune diseases. Repurposing these drugs, following approval from regulatory bodies (Table 1), has the potential as a new therapy for SARS‐CoV‐2‐induced ARDS, in which the cytokine storm plays an immunopathological role. This minireview includes anti‐cytokine drugs tested in COVID‐19 patients, focusing on IL‐1β, IL‐4/IL‐13, IL‐6 and TNF‐α. Case studies and studies focused primarily on anti‐cytokine treatment in other diseases, such as psoriasis, in which a small number of participants contracted COVID‐19 during the study, were omitted because of difficulties in drawing firm conclusions based on such studies. This minireview also focuses on clinical trials that have already been completed with available study data. Emphasis is given to drugs tested in randomised controlled trials (RCTs), where available, than observational studies. A large number of further studies are currently underway, some of which are summarised in Table 1. In several studies, a significant proportion of the patients had one or more co‐morbidities. However, this information was not included in each investigation unless it had considerably impacted the study conclusions.

**TABLE 1:** Clinical trials of anti‐cytokine drugs in COVID‐19 patients

Target cytokines	Drug name (brand name)	Mechanism	Clinical trials (status)	Results
IFN‐γ	Emapalumab (Gamifant)—Treatment of haemophagocytic lymphohistiocytosis	Human monoclonal antibody (mAb) binds to IFN‐γ and blocks ligand‐receptor interaction	NCT04324021 (terminated)	Not available
TNF‐α	Infliximab (Remicade)—Treatment of ulcerative colitis, paediatric ulcerative colitis, psoriatic arthritis, ankylosing spondylitis, Crohn's disease, paediatric Crohn's disease, plaque psoriasis and rheumatoid arthritis	Chimeric mAb targeting TNF‐α—Functions by acting as a decoy receptor to TNF‐α which prevents it from binding to its receptor and in turn, neutralising the activity of TNF‐α	NCT04425538 (completed) NCT04593940 (completed) NCT04734678 (recruiting) NCT04344249 (recruiting) NCT04922827 (recruiting) NCT05220280 (not yet recruiting)	NCT04425538 (observational)—Improvement in oxygen saturation. Infliximab rapidly abrogates inflammatory signalling to facilitate clinical recovery in severe and critical COVID‐19 patients.
	Adalimumab (Humira)—Treatment of rheumatoid arthritis, juvenile idiopathic arthritis, psoriatic arthritis, ankylosing spondylitis, Crohn's disease and plaque psoriasis	Human mAb targeting TNF‐α—Specifically targets TNF‐α and works by blocking its interaction with the p55 and p75 cell surface TNF receptors	NCT05014555 (not yet recruiting) ChiCTR2000030089 (suspended) NCT04705844 (withdrawn)	Not available
IL‐1β	Anakinra (Kineret)—Treatment of rheumatoid arthritis and neonatal‐onset multisystem inflammatory disease	An IL‐1 receptor (IL‐1R) antagonist	NCT04680949 (active, not recruiting)^a^ NCT04341584 (completed) NCT04318366 (recruiting)^a^ NCT04443881 (completed) NCT04362943 (completed) NCT04339712 (completed) NCT04362111 (active, not recruiting) NCT04594356 (active, not recruiting) NCT04412291 (recruiting) NCT04357366 (recruiting) NCT04643678 (recruiting) NCT04408326 (recruiting) NCT04148430 (recruiting) NCT04381936 (recruiting) NCT02735707 (recruiting) NCT04278404 (recruiting) NCT05279391 (recruiting) NCT04424056 (not yet recruiting) NCT04603742 (withdrawn) NCT04462757 (terminated) NCT04366232 (terminated) NCT04364009 (terminated) NCT04324021 (terminated) NCT04374539 (terminated)	NCT04680949 (RCT)—Treatment with anakinra in severe COVID‐19 patients significantly reduced the risk of worse clinical outcomes. Inflammatory markers were significantly decreased. NCT04341584 (RCT)—Treatment with anakinra in COVID‐19 patients (mild to severe) did not improve clinical outcomes. NCT04318366 (observational)—Treatment with high‐dose anakinra was safe and associated with reductions in CRP, improvements in respiratory function and survival.
	Canakinumab (Ilaris)—Treatment of cryopyrin‐associated periodic syndromes, familial Mediterranean fever and active systemic juvenile idiopathic arthritis.	Human mAb that binds to IL‐1β and blocks its interaction with IL‐1 receptors.	NCT04362813 (completed) NCT04365153 (completed) NCT04510493 (completed) NCT04278404 (recruiting) NCT05080218 (not yet recruiting) NCT04348448 (unknown) NCT04476706 (no longer available)	NCT04362813 (RCT)—No significant improvements in the likelihood of survival. However, CRP, ferritin, and D‐dimer levels were reduced in patients treated with canakinumab.
IL‐2	Basiliximab (Simulect)—Treatment of donor kidney transplantation recipients to prevent immune rejection of transplanted kidney.	Mouse‐human chimeric mAb that is directed at the alpha‐subunit of the IL‐2 receptor (CD25), which blocks IL‐2 binding.	NCT05013034 (not yet recruiting)	Not available
IL‐4 and IL‐13	Dupilumab (Dupixent)—Treatment for atopic dermatitis, asthma, and nasal polyps accompanied by chronic rhinosinusitis	Human monoclonal antibody of IgG4 subclass. Inhibits IL‐4 and IL‐13 expression through blockage of IL‐4Rα	NCT04920916 (active, not recruiting)	NCT04920916 (RCT)—Treatment with dupilumab in COVID‐19 patients saw reduced ICU admission and reduced mortality compared with placebo patients.
IL‐6	Tocilizumab (Actemra)—Treatment of rheumatoid arthritis, polyarticular juvenile idiopathic arthritis, giant cell arthritis, systemic juvenile idiopathic arthritis and cytokine release syndrome	mAbs against IL‐6 receptor and bind to both soluble and membrane‐bound IL‐6 receptors	NCT02735707 (recruiting)^a^ NCT04372186 (active, not recruiting) NCT04381936 (recruiting)^a^ NCT04423042 (not yet recruiting)^a^ NCT04347993 (active, not recruiting)^a^ NCT04445272 (completed) NCT04730323 (completed) NCT04331795 (completed) NCT04600141 (completed) NCT04893031 (completed) NCT04678739 (completed) NCT04320615 (completed) NCT04363736 (completed) NCT04873141 (completed) NCT04409262 (completed) NCT04356937 (completed) NCT04577534 (completed) NCT04690920 (completed) NCT04339712 (completed) NCT04519385 (completed) NCT04330638 (completed) NCT04492501 (completed) NCT04476888 (completed) NCT04454372 (completed) NCT04347031 (completed) NCT04349410 (completed) NCT04854941 (completed) NCT04346693 (completed) NCT05302947 (completed) NCT04479358 (recruiting) NCT05082714 (recruiting) NCT04412772 (recruiting) NCT05164133 (recruiting) NCT04332094 (recruiting) NCT04924829 (recruiting) NCT04359667 (recruiting) NCT04412291 (recruiting) NCT04560205 (recruiting) NCT04335305 (recruiting) NCT04734678 (recruiting) NCT05118737 (recruiting) NCT04779047 (recruiting) NCT04871854 (recruiting) NCT04693026 (recruiting) NCT05017441 (recruiting) NCT04476979 (recruiting) NCT04486521 (recruiting) NCT04380818 (recruiting) NCT04681040 (recruiting) NCT04826588 (recruiting) NCT04351503 (recruiting) NCT04278404 (recruiting) NCT05279391 (recruiting) NCT04321993 (recruiting) NCT04317092 (active, not recruiting) NCT04377659 (active, not recruiting) NCT04363853 (active, not recruiting) NCT05002517 (active, not recruiting) NCT05057962 (active) NCT05035589 (not yet recruiting) NCT04361032 (not yet recruiting) NCT04424056 (not yet recruiting) NCT04536363 (not yet recruiting) NCT04345445 (unknown) NCT04377750 (unknown) NCT04310228 (unknown) NCT04306705 (unknown) NCT04332913 (unknown) NCT04315480 (unknown) NCT04331808 (unknown) NCT04394182 (suspended) NCT04335071 (terminated) NCT04435717 (terminated) NCT04346355 (terminated) NCT04377503 (terminated) NCT04403685 (terminated) NCT04322773 (terminated) NCT04370834 (terminated) NCT04374539 (terminated) NCT05133635 (withdrawn) NCT04361552 (withdrawn)	NCT02735707 (RCT)—Patients treated with tocilizumab showed reduced mortality and faster recovery than nontreated patients. NCT04372186 (RCT)—Compared with the placebo group the use of tocilizumab reduced the likelihood of progression to the composite outcome of mechanical ventilation or death, but it did not improve survival in COVID‐19 pneumonia patients. NCT04381936 (RCT)—Tocilizumab can improve survival and clinical outcomes in COVID‐19 patients with hypoxia and systemic inflammation
	Siltuximab (Sylvant)—Treatment of multicentric Castleman's disease in non‐HIV patients	Mouse‐human chimeric mAb—Functions by binding to IL‐6 to prevent interaction with its receptor	NCT04322188 (completed) NCT04486521 (recruiting) NCT04329650 (unknown)	NCT04322188 (observational)—Treatment with siltuximab was associated with reduced mortality, CRP levels and cytokine‐driven hyperinflammation.
	Sarilumab (Kevzara)—Treatment of rheumatoid arthritis	mAbs against IL‐6 receptor and bind to both soluble and membrane‐bound IL‐6 receptors	NCT02735707 (recruiting)^a^ NCT04318366 (recruiting)^a^ NCT04357860 (completed) NCT04327388 (completed) NCT04315298 (completed) NCT04357808 (completed) NCT04359901 (active, not recruiting) NCT04386239 (recruiting) NCT04278404 (recruiting) NCT04661527 (unknown) NCT04324073 (unknown) NCT04341870 (suspended) NCT04322773 (terminated)	NCT02735707 (RCT)—Patients treated with sarilumab showed reduced mortality and faster recovery than nontreated patients. NCT04318366 (observational)—Clinical improvement and mortality in severe COVID‐19 patients did not show significant differences between sarilumab and standard of care. However, the survival rate reported in patients treated with sarilumab was higher than in the control group.
IL‐12/23	Risankizumab (Skyrizi)—Treatment of plaque psoriasis	Human mAb selective for IL‐23 and specifically inhibits the interaction of IL‐23 with its receptor by targeting the p19 alpha subunit of IL‐23	NCT04583956 (completed)	Not available
	Ustekinumab (Stelara)—Treatment of plaque psoriasis, psoriatic arthritis, Crohn's disease and ulcerative colitis	Human mAb directed against IL‐12 and IL‐23 and binds to the common p40 subunit shared by IL‐12 and IL‐23. Its binding to the p40 subunit prevents IL‐12 and IL‐23 from binding to their respective receptors, IL‐12Rβ1/β2 and IL‐12Rβ1/23R, inhibiting their biological activity	NCT05014555 (not yet recruiting)	
IL‐17A	Secukinumab (Cosentyx)—Treatment of uveitis, rheumatoid arthritis and ankylosing spondylitis	Human mAb that binds to IL‐17A and inhibits its interaction with the IL‐17 receptor	NCT04403243 (unknown) NCT05080218 (not yet, recruiting)	
	Ixekizumab (Taltz)—Treatment of severe plaque psoriasis and psoriatic arthritis	Humanised immunoglobulin G subclass 4 (IgG4) mAb—Acts as a soluble decoy receptor that binds to IL‐17A preventing its interaction with the IL‐17 receptor	NCT04724629 (completed)	
IL‐6 and IL‐1β	Anakinra, tocilizumab, siltuximab		NCT04330638 (completed)	NCT04330638 (RCT)—Treatment with anakinra in COVID‐19 patients with hypoxia did not improve clinical outcomes. No significant differences were observed in median time to clinical improvement in COVID‐19 patients. However, clinical improvement was noted in patients treated with siltuximab.

Abbreviations: CRP; C‐reactive protein; RCTs; randomised controlled trials.

**Information on clinical trials status:** **Active, not recruiting:** The study is ongoing, and participants are receiving an intervention or being examined, but potential participants are not currently being recruited or enrolled. **Withdrawn:** The study stopped early, before enrolling its first participant. **Unknown:** A study on ClinicalTrials.gov whose last known status was recruiting; not yet recruiting; or active, not recruiting but that has passed its completion date, and the status has not been last verified within the past 2 years. **Suspended:** The study was stopped early, but it may resume at a later date.

^a^ Despite the availability of results, clinicaltrials.gov might not have updated the status.

## 2 IL‐1β

### 2.1 Anakinra

A double‐blinded, randomised, controlled, phase 3 trial at 37 study sites in Greece and Italy (NCT04680949) recruited 594 patients with moderate or severe COVID‐19 who were at risk of progressing to respiratory failure based on soluble urokinase plasminogen activator receptor (a biomarker that predicts the development of severe COVID‐19) serum levels of ≥6 ng ml^−1^. A total of 189 patients were assigned to the placebo group (mean age, 61.5 years) and 405 to the treatment group (mean age, 62 years; 100‐mg anakinra administered once daily for 10 days). Overall, 50.4% (204/405) of patients treated with anakinra experienced full recovery with no viral RNA detected on day 28 compared with 26.5% (50/189) in the placebo group. Patients treated with anakinra had a higher lymphocyte count and lower levels of IL‐6 and C‐reactive protein (CRP), an acute phase inflammatory marker, than those in the placebo group (Kyriazopoulou et al., 2021). The mortality rate in the treatment group was lower (3.2%; 13/405) than that (6.9%; 13/189) in the placebo group. The frequency of treatment‐emergent adverse events, such as infections, ventilator‐associated pneumonia, septic shock and multiple organ dysfunction, was higher (21.7%; 41/189) in the placebo group than that (16%; 65/405) in the treated group. Thus, anakinra treatment in patients with moderate or severe COVID‐19 leads to improved clinical outcomes.

In a multicentre, open‐label, randomised trial in France (NCT04341584), 116 COVID‐19 patients with moderate to severe pneumonia (not requiring mechanical ventilation) or critical pneumonia (requiring mechanical ventilation) were recruited from 16 hospitals. Fifty‐nine patients (age 55–74 years; median age, 67 years) received anakinra treatment (200 mg twice per day on days 1–3, 100 mg twice on day 4, and 100 mg once on day 5), while 55 (age 59–78 years; median age, 65 years) received standard care treatment. Requirement of non‐invasive or mechanical ventilation or death, was observed in 28/59 patients that received anakinra treatment and in 28/55 patients of the standard treatment group. Thirty‐eight patients from the anakinra‐treated and 41 from the standard treatment group died. Between the two groups, there were no differences in the levels of acute phase inflammatory markers (C‐reactive protein, ferritin or lactate dehydrogenase [LDH]) or in the number of patients who were relieved of oxygen support and discharged. This study showed that anakinra did not improve outcomes in COVID‐19 patients (Corimuno‐Collaborative‐Group, 2021).

In a retrospective cohort, observational study at the San Raffaele Hospital in Milan, Italy (NCT04318366), patients with moderate‐to‐severe COVID‐19 and experiencing ARDS with bilateral infiltrates as well as hypoxemia were assigned to two groups:‐ 29 patients (age 55–71 years; median age, 62 years) were administered a high dose of anakinra (5 mg·kg^−1^ body weight, twice daily; median duration of treatment, 9 days), while 16 patients (age 64–78 years; median age, 70 years) received standard treatment. At 21 days, patients in the anakinra treatment group showed reductions in serum C‐reactive protein and improvements in respiratory function (72%, 21/29) and survival (90%, 26/29). Of the eight patients in the anakinra‐treated group that did not show any improvement, five underwent mechanical ventilation, and three died. In the standard treatment group, 50% (8/16) of the patients showed improved respiratory function, while amongst the other eight patients, one underwent mechanical ventilation and seven (44%) died (Cavalli et al., 2020). In summary, this study found that anakinra treatment led to clinical improvement in more than 70% of patients.

#### 2.1.1 Overall evaluation

The two randomised controlled trials described above reached different conclusions about the value of anakinra treatment in COVID‐19. The French study used a 5‐day anakinra treatment protocol, which was significantly shorter than the treatment in studies conducted in Greece and Italy (NCT04680949, NCT04318366). This may explain why anakinra treatment failed to improve COVID‐19 in those patients. Further studies are required, but the positive outcomes reported by Kyriazopoulou et al. provide clear grounds for optimism, mainly when considered with the promising observational data of Cavalli et al. There may also be a pharmacogenomic effect associated with the drug because it was previously reported that rheumatoid arthritis (RA) patients with IL‐1α + 4,845(G > T) polymorphism may respond better to anakinra (Camp et al., 2005). A pharmacogenomic effect, however, was not considered in these studies.

### 2.2 Canakinumab

A randomised, double‐blind, placebo‐controlled phase 3 trial (NCT04362813) recruited 454 patients with severe COVID‐19 pneumonia and hypoxia (not requiring invasive mechanical ventilation) from 39 hospitals in Europe and the United States to test the efficacy of canakinumab. Patients were divided into two groups evenly; 223 patients (age 49–69 years; median age, 59 years) were administered canakinumab (single dose, 450 mg for body weight 40 to <60 kg, 600 mg for 60–80 kg, and 750 mg for >80 kg), while the other 223 (age 50–68 years; median age, 57 years) received a placebo. Eighty patients in the canakinumab‐treated and 68 patients in the placebo group experienced complete recovery. Patients requiring mechanical ventilation were 20 and 29 in the canakinumab‐treated and placebo groups, respectively. The morbidity rate in canakinumab‐treated patients was 88.8% (25/223) compared with 85.7% (32/223) in the nontreated ones. Twelve patients from the canakinumab‐treatment group, and 19 from the placebo group, remained hospitalised till day 29 post‐treatment (Caricchio et al., 2021). Thus, canakinumab treatment did not significantly increase the likelihood of survival without the requirement of invasive mechanical ventilation.

In a single‐centre cohort observational study conducted at the Annunziata Hospital of Chieti, Italy, 34 patients with mild or severe non‐intensive care unit (ICU) COVID‐19 were enrolled and split equally into a treatment group (age 48–62 years; median age, 53 years) that was administered canakinumab (single dose of 300 mg), and a control group (age 50–72; median age, 59) that underwent standard therapy. Canakinumab‐treated patients showed improved lymphocyte and platelet counts and reduced levels of C‐reactive protein and LDH compared with nontreated patients. A significant increase in the arterial oxygen partial pressure to fractional inspired oxygen (PaO_2_:FiO_2_) ratio was observed in patients administered canakinumab (60.3%) compared with patients who were not (39.2%). No deaths were reported in either group (Katia et al., 2021). The efficacy of canakinumab treatment was further assessed in another retrospective analysis at the same hospital, involving 10 COVID‐19 patients (age 44–66 years; median age, 51 years) with severe symptoms such as bilateral pneumonia, hyperinflammation, and respiratory failure. In canakinumab‐treated (single dose of 300 mg) patients, C‐reactive protein levels decreased by 80.9% (day 3) to 99% (day 7) from the baseline, while in nontreated patients, the levels increased by 25.8% (day 3) and then decreased by 29.0% (day 7). The PaO_2_:FiO_2_ ratio increased from the baseline by 28.5% (day 3) to 41.4% (day 7) in the treatment group, whereas in the nontreated group, the ratio increased by 1.7% (day 3) to 38.8% (day 7). No deaths were reported in canakinumab‐treated patients, compared with one death and the requirement of oxygen therapy by one patient in the untreated group (Ucciferri et al., 2020). In summary, canakinumab treatment led to a rapid reduction in systemic inflammation and improved oxygenation.

#### 2.2.1 Overall evaluation

Although canakinumab treatment did not lead to significant overall clinical improvement, it helped reduce inflammation and improve oxygenation in COVID‐19 patients. The anti‐inflammation effect of canakinumab suggests its potential use as an adjuvant drug in COVID‐19 treatment. Caricchio et al. found that canakinumab had no major influence on the course of COVID‐19 infection, while Ucciferri et al. found it resulted in a rapid reduction in systemic inflammation. We note that 454 patients were enrolled in the study by Carrichio et al., while Ucciferi et al. had a much smaller patient cohort (n = 34). Thus, the weight of evidence suggests that canakinumab has little or no benefit in COVID‐19, although the results of Ucciferri et al. suggest that further studies are warranted.

## 3 IL‐4/IL‐13

### 3.1 Dupilumab

A randomised, double‐blinded, placebo‐controlled trial (NCT04920916) was carried out in Virginia showed that patients treated with dupilumab had reduced mortality outcomes (10.5%, 2/19 patients) compared with placebo patients (23.8%, 5/21 patients) at day 60. Patients were split into placebo group (n = 21; age 55–78; median age 63) and dupilumab‐treated group (n = 19; age 44–70; median age 59; administered with two doses of 300 mg, with a follow up of 300 mg maintenance dose on day 14 or 28 if patients remained hospitalised). Ferritin, C‐reactive protein, and IgE levels were comparable between placebo and dupilumab groups. Eosinophil levels were shown to be elevated in patients receiving dupilumab compared with patients in the placebo group. Patients in the placebo group had a higher probability of ICU admission compared with patients in the dupilumab group.

#### 3.1.1 Overall evaluation

The results of this study showed some advantages of dupilumab as a treatment option for COVID‐19 (Sasson et al., 2022). However, this study was limited by the small size of the cohort and the potential adverse effects of dupilumab, such as hyper‐eosinophilia. Based on the findings of this initial investigation, further research is needed.

## 4 IL‐6

### 4.1 Tocilizumab

A randomised, double‐blind, placebo‐controlled trial was conducted in 51 hospitals in Brazil, Kenya, Mexico, Peru, South Africa and the United States to test the safety and efficacy of tocilizumab in treating COVID‐19 (NCT04372186). A total of 377 COVID‐19 patients who were not receiving mechanical ventilation were involved in the study, with 249 patients (mean age 56.0 ± 14.3) receiving standard care plus one or two doses of tocilizumab (8 mg·kg^−1^ of body weight) and 128 patients (mean age 55.6 ± 14.9) received standard care plus placebo. By day 28, 12% of the patients in the tocilizumab‐treatment group and 19.3% in the placebo group received mechanical ventilation or died. Time‐to‐event analysis favoured tocilizumab over placebo. The study indicated that tocilizumab plus standard care could reduce the likelihood of the composite outcome of progression to mechanical ventilation or death (Salama et al., 2021).

A randomised, controlled, open‐label platform trial was conducted in Ghana, India, Indonesia, Nepal, South Africa, Sri Lanka, the United Kingdom and Vietnam to assess tocilizumab's efficacy in COVID‐19 patients (NCT04381936). The study involved 4116 COVID‐19 patients with 2022 in the tocilizumab‐treatment group (mean age 63.3 ± 13.7) and 2094 in the usual care group (mean age 63.9 ± 13.6). Patients received tocilizumab at 800 mg if weight >90 kg; 600 mg if weight >65 and ≤90 kg; 400 mg if weight >40 and ≤65 kg; and 8 mg·kg^−1^ if weight ≤40 kg. Within 28 days, 621 patients (31%) in the tocilizumab‐treatment group and 729 (35%) patients in the usual care group died. The median time to discharge from the hospital is >28 days for the usual care group and 19 days for the tocilizumab‐treatment group. The study demonstrated that tocilizumab could improve survival and other clinical outcomes, such as mechanical ventilation and the use of haemodialysis or haemofiltration (RECOVERY Collaborative Group, 2021).

### 4.2 Tocilizumab and sarilumab

A multifactorial, adaptive platform trial was conducted in the United States, United Kingdom and France to evaluate the efficacy and safety of tocilizumab and sarilumab (NCT02735707). A total of 895 COVID‐19 patients were admitted to the ICU and received respiratory or cardiovascular organ support. Of these, 353 patients (mean age, 61.5 ± 12.5) were assigned to the tocilizumab group (administered 8 mg·kg^−1^ body weight tocilizumab, going up to 800 mg; repeat dose administered to 29% of patients at the volition of the treating physician), 48 (mean age 63.4 ± 13.4) to the sarilumab group (administered a single dose of 400 mg sarilumab), 402 (mean age 61.1 ± 12.8) to the control group (no treatment) and 69 to the group undergoing other interventions. The median numbers of organ support‐free days were 10, 11 and 0 in the tocilizumab, sarilumab and control groups, respectively. Tocilizumab treatment had no significant effect on PaO_2_, FiO_2_, C‐reactive protein or D‐dimer levels. Death was reported in 28% (98/350), 22% (10/45), and 36% (142/397) of the patients in the tocilizumab, sarilumab and control groups, respectively. An analysis of the 90‐day survival rate showed improved survival in pooled IL‐6 receptor antagonist groups (Gordon et al., 2021).

### 4.3 Combination treatment (anakinra, tocilizumab, siltuximab)

A prospective, multicenter, open‐label, randomised, controlled clinical trial in Belgium (NCT04330638) compared the safety and effectiveness of individually or simultaneously blocking IL‐6 and IL‐1 versus standard of care, on blood oxygenation and systemic cytokine release syndrome, in patients with COVID‐19 and acute hypoxic respiratory failure. A total of 342 COVID‐19 patients were randomly assigned to anakinra treatment (n = 112; age 56–74 years; median age 67 years; administered 100 mg anakinra once per day until day 28 or discharge) or no anakinra treatment (n = 230; age 54–72 years; median age 64 years), and simultaneously randomly assigned to IL‐6 blockade (n = 227; age 54–73 years; median age 65 years; 114 for tocilizumab: a single dose of 8 mg·kg^−1^ body weight, and 113 for siltuximab: a single dose of 11 mg·kg^−1^ body weight) or no IL‐6 blockade (n = 115; age 55–72 years; median age 64). This study did not show any effect of the treatments on the number of deaths, acute phase reactants, or median time to clinical improvement in COVID‐19 patients (Declercq et al., 2021).

#### 4.3.1 Overall evaluation

These studies highlight tocilizumab as a promising therapeutic agent in COVID‐19. Tocilizumab has been approved for provisional usage to treat COVID‐19 by the European Medical Agency (EMA), Food and Drug Administration (USA) and the Australian Therapeutic Goods Administration (TGA). It is intriguing that simultaneously blocking IL‐6 and IL‐1 provided no discernible clinical benefit. The underlying mechanism involved in this outcome is not clear. One possibility to consider is that pharmacogenetics could influence study outcomes. The IL‐6R rs12083537 (A > G) polymorphism has been associated with tocilizumab efficacy (Janahiraman et al., 2022). It is possible that genetic variations in IL‐6R may improve the prediction of tocilizumab therapy outcomes in COVID‐19 patients (Badary, 2021). However, this factor was not taken into account in these studies. A wide variety of additional variables could also contribute to the lack of effect following simultaneous IL‐1/IL‐6 blockade, such as disease severity or patient age.

## 5 TNF‐α

### 5.1 Infliximab

In a prospective, single‐centre phase 2 trial at Tufts Medical Centre in Boston (NCT04425538), infliximab treatment (5 mg·kg^−1^ body weight, retreatment at physician discretion between 7–21 days following the first treatment) in 18 adults (age 31–80 years; median age, 63 years) with severe COVID‐19 (hypoxic respiratory failure and pneumonia) resulted in 89% (16/18) of patients showing a sustained improvement in the ratio of oxygen saturation to the fraction of inspired oxygen (SpO_2_/FiO_2_), and 83% (15/18) having a resolution of respiratory failure in room air or following nasal cannula oxygen supplementation. One patient on extracorporeal membrane oxygenation was successfully decannulated after 16 days. There were 15 survivors and three deaths at the end of the 28‐day study period, with the cause of death being secondary lung infection. Key proinflammatory cytokines such as IL‐6, TNF‐α, IFN‐γ and IP‐10 (CXL‐10), as well as ferritin and C‐reactive protein, showed a rapid decline from elevated baseline levels (Hachem et al., 2021). These results indicate that infliximab blocks inflammatory signalling and promotes recovery in patients with severe COVID‐19.

In a small cohort of seven critically ill COVID‐19 patients (age 47–70 years; median age, 60 years) with severe symptoms (cytokine storm syndrome along with organ failure) at the Jena University Hospital in Germany, infliximab treatment (5 mg·kg^−1^ body weight between day 0 and 3 following admission) led to a rapid but temporary decrease in IL‐6, C‐reactive protein and LDH. Six of the seven patients showed improved clinical symptoms, and five showed elevated lymphocyte counts after treatment. However, one death was observed in the infliximab‐treated group. The deceased had shown severe procoagulant activity with repeated episodes of fulminant thromboembolic events in the pulmonary circulation and highly upregulated ferritin levels before infliximab treatment. A separate group of 17 patients (age 42–91 years; median age, 66 years) diagnosed with severe COVID‐19 and undergoing supportive therapy such as oxygen supplementation, was selected retrospectively as a control group for the infliximab‐treated group. Of these, six patients (35%) showed overall mortality at a similar hospitalisation stage and prolonged systemic inflammation (Stallmach et al., 2020). Despite the limited sample size, these findings support the potential of infliximab as a therapeutic agent against SARS‐CoV‐2.

#### 5.1.1 Overall evaluation

To date, no randomised controlled trials data have been published for drugs that target TNF‐α. Given the importance of TNF‐α cytokine in COVID‐19 disease, we have presented the results of two observational studies above. These results are promising and we await randomised controlled trials data with some optimism.

## 6 CONCLUSIONS

Antiviral drugs targeting SARS‐CoV‐2, including Paxlovid® (nirmatrelvir + ritonavir) and molnupiravir, have recently been developed (Burki, 2022). These are the first oral medications to be approved for treating mild to moderate COVID‐19 and have been used to treat individuals at a high risk of severe disease (Fishbane et al., 2022; Tian et al., 2022). This minireview has highlighted the promising data indicating that anti‐cytokine drugs are also likely to have value in treating COVID‐19. The way forward may be to use a combination of antivirals and anti‐cytokine drugs to reduce morbidity and mortality in patients with COVID‐19.

### 6.1 NOMENCLATURE OF TARGETS AND LIGANDS

Key protein targets and ligands in this article are hyperlinked to corresponding entries in the IUPHAR/BPS Guide to PHARMACOLOGY http://www.guidetopharmacology.org and are permanently archived in the Concise Guide to PHARMACOLOGY 2021/22 (Alexander et al., 2021).

**Abbreviations**
ARDS: acute respiratory distress syndrome
CoV: coronavirus
FiO_2_
: fractional inspired oxygen
MEK1/2: mitogen‐activated protein kinase
PaO_2_
: arterial oxygen partial pressure

## CONFLICTS OF INTEREST

The authors declare no conflicts of interest.

## AUTHOR CONTRIBUTIONS

**Wern Hann Ng:** Writing‐original draft (lead); writing‐review and editing (lead). **Patrick Chun Hean Tang:** Writing‐original draft (supporting); writing‐review and editing (supporting). **Suresh Mahalingam:** Conceptualization (equal); supervision (equal); writing‐original draft (lead); writing‐review and editing (lead). **Xiang Liu:** Supervision (equal); writing‐original draft (lead); writing‐review and editing (lead).

## Data Availability

N/A‐Review.
